# Associated Effects and Efficiency Evaluation between Wastewater Pollution and Water Disease Based on the Dynamic Two-Stage DEA Model

**DOI:** 10.3390/healthcare8030279

**Published:** 2020-08-19

**Authors:** Ya-nan Sun, Fang-rong Ren, Jia-wei Liu, Nai-xin Shi

**Affiliations:** 1 Economics and Management School, Nantong University, Nantong 226019, China; sun.yn@ntu.edu.cn (Y.-n.S.); 18361136675@163.com (J.-w.L.); Shi990926@163.com (N.-x.S.); 2 Business School, Hohai University, Nanjing 211100, China

**Keywords:** dynamic network DEA, efficiency, wastewater pollution, water diseases

## Abstract

The lack of basic water supply and treatment facilities during China’s urbanization and industrialization process has resulted in a large amount of wastewater pollution, with the most serious water diseases being water-borne endemic fluorosis and arsenic poisoning, which have affected more than 20 million people. This research therefore uses the improved modified undesirable dynamic network model to analyze data of 31 provincial administrative regions to focus on the associated effects and efficiency evaluation between wastewater pollution and water disease in China. The results show that the efficiency of water pollution disease in all four regions of the country and the total efficiency in the east, west, and central regions all show a decreasing trend, while the efficiency scores and rankings of all provinces and cities within the region fluctuate greatly. The eastern region with the most developed economy has the best overall performance, with higher efficiency in water consumption and water disease control. However, the efficiency of wastewater treatment in northeast China is stable and better. Given the high level of the nation’s economic development and the results of efficiency in water pollution and water diseases, improving the efficiency and quality of wastewater treatment in China is regarded as an important factor for achieving the strategic goal of green growth.

## 1. Introduction

Water is the basic requirement for maintaining life and health, and although 70.8% of the earth is covered by water, freshwater resources are still extremely limited [1]. In the face of increasing demand, water issues are a top priority to resolve for any country targeting economic growth. At present, China’s social and economic development ranks at the forefront of the world, but its water shortage problem is very serious. At the end of 2018, the country had total water resources of 2796 billion cubic meters or 2004 cubic meters per person, taking up one quarter of the world’s average [2]. China is one of 13 water-poor countries in the United Nations, especially in the north and parts of the east where per capita water resources are seriously low [3]. The regional distribution of water resources per capita in China is also inversely proportional to the level of regional economic development. In the economically developed Beijing-Tianjin-Hebei region, per capita water resources are less, while in the economically underdeveloped southwest region, per capita water resources exceed the national average level. However, with continuous development of its economy and the increasing living standard of residents, water consumption continues to be very high, with total water consumption of 602.12 billion cubic meters in 2019 [4]. The direct imbalance between demand and supply of water resources leads to a large water shortage in the eastern developed regions of China, which are facing a more serious water shortage problem.

Another situation that is more mismatched than water shortages is the serious problem of water pollution in China. In 2017, China’s total wastewater discharge was 69.97 billion tons: industrial wastewater discharge at 18.16 billion tons, or 26.0% of total emissions, and urban domestic sewage discharge at 51.78 billion tons, or 74.0% of total discharge [5]. The proportion of urban domestic sewage is increasing year by year and is the main source of sewage. In 2017, the “Bulletin on the Circumferential Situation of Ecology in China” reported 940 surface water quality sections in which the proportion of inferior V-type water quality is 8.3% (The quality of inferior V-type water is worse than V-type water quality, and detailed classification criteria are in the Appendix A), and among the 5100 groundwater quality monitoring points the proportion of poorer and lower points is 66.6%. Two-thirds of China’s cities are already facing water shortages, and the already limited clearwater resources are being destroyed by discharged sewage, further exacerbating water scarcity.

As water pollution worsens, human health also faces a serious threat. Approximately 80% of the global population is threatened by water shortages, mainly caused by water pollution, overuse of water, and climate change [6]. Water pollution accounting for two million deaths per year has become a major global problem, because of its association with numerous diseases (e.g., salmonella, typhoid, skin infections, trachoma, cancer, cholera, and polio) [7,8,9]. In China, only 550 million residents, accounting for 39.28% of the total population, have seen their drinking water safety improved [10].

As early as 1999, Wu et al. [11] pointed out that the process of urbanization and industrialization in China has brought about tremendous pollution, coupled with inadequate investment in basic water supply and treatment infrastructure, resulting in extensive wastewater pollution. The extreme waste of water resources poses a challenge to sustainable development, depleting energy reserves and destroying humans’ water security and ecosystem health. The China government has attached great importance to the predicament of water pollution. In 2011, 2015, and 2017, it respectively formulated the National Groundwater Pollution Prevention and Control Plan (2011–2020), the Water Pollution Prevention Action Plan, and the Key Basin Water Pollution Prevention and Control Plan (2016–2020) [12] and also put forward the development concept of prioritizing saving water, strengthening water resources management, and controlling water pollution to the greatest extent. Therefore, in order to solve the shortcomings of static analysis, regional differences, and health factors, our research proposes a modified undesirable dynamic network model to explore economic, wastewater treatment, and human health efficiencies of 31 provincial-level administrative regions in China.

To sum up, the present literature on water pollution, wastewater treatment, and water diseases mainly presents the following types: (1) economic and feasibility studies on wastewater treatment [13,14,15], (2) research on wastewater treatment and health [6,16], and (3) research on the health effects of air pollution [17,18,19,20]. Among the economic and feasibility studies of wastewater treatment and wastewater treatment plants, most studies focused on the environmental performance indicators (EPIs), cost efficiency of wastewater treatment plants, as well as their environmental benefits analysis [13,21,22,23]. They used research methods such as shadow price, life cycle assessment (LCA), life cycle costing (LCC) [15,24], cost–benefit analysis (CBA) [14,22,23], and data envelopment analysis (DEA) [25,26,27,28,29,30]. Among them, DEA is widely used as a kind of efficiency evaluation method. Its advantage is that it can compare multiple decision-making units and can flexibly select the input–output index according to the characteristics of the evaluation object, so as to establish an evaluation index system more in line with analysis needs.

There is more and more empirical research on public health problems caused by environmental problems, but most existing literature focuses on the consequences of air pollution such as mortality [18,31,32], public health [17,19] and individual health of employees [33,34], and the impact of air pollution on government expenditures and personal medical expenditures [20,35]. In the study of water pollution, however, most studies target infant and child mortality [18,21], while few discuss a combined analysis of the economy, water pollution, and health from a comprehensive perspective.

This research offers the following three innovations and contributions. First, most current studies do not discuss the economy, water pollution, and public health together. In this paper, we study economic, wastewater discharge, and wastewater pollution efficiencies, explore the government’s wastewater treatment input, and water disease efficiency. Compared with a general study on environmental pollution and health efficiency [36], this study is more focused on water diseases caused by the discharge and treatment of wastewater pollution so as to promote water resource management and water pollution control to the maximum extent in China. In particular, we select the two most representative types of endemic water diseases, water fluorosis and arsenic poisoning, as the subjects to fill the gap in the research on the differences in the prevention and control efficiency of water diseases among different regions.

Second, the modified undesirable dynamic network model can avoid the shortcomings and problems of previous static analysis. This is an important improvement of the empirical model for an efficiency evaluation of the DEA technique in two different stages. It can also enlighten and help subsequent scholars to carry out further research on multi-stage efficiency evaluation and DEA model improvement.

Third, the variable selection of associated effects and efficiency evaluation between wastewater pollution and water disease is more comprehensive and detailed. In this study, the production stage is Stage 1, and the health stage is Stage 2. The inputs of Stage 1 are production stage labor and water consumption, while the outputs of Stage 1 are GDP and wastewater. The variable that links the production stage and health stage is chemical oxygen demand (COD). The input of Stage 2 is wastewater treatment expense, the outputs of Stage 2 are wastewater treatment capacity and number of water diseases, and the carry-over variable is fixed assets.

## 2. Method and Model

### 2.1. SBM Dynamic Network DEA

The weighted slack-based measures (SBM) network DEA model was set up by Tone and Tsutsui [37] in 2009. In this model, each department is regarded as a sub-DMU (Decision Making Unit). A linkage among departments of decision-making units can be used as the analysis basis of the network DEA model. The network DEA model improves the part of the traditional DEA that fails to analyze the performance of each department.

Tone and Tsutsui [38] in 2013 put forward the weighted SBM dynamic network DEA model by adding carry-over activities. As a form of linkage, the carry-over activities can be divided into four categories: (1) Desirable. This is an output that is beneficial to the overall objective and in line with expectations, in which a greater value is better. (2) Undesirable. This is an output that is unhelpful to the overall objective and does not meet expectations, in which a smaller value is better. (3) Discretionary. (4) Non-discretionary.

### 2.2. The Modified Undesirable Dynamic Network Model

This study utilizes panel data (Also known as “parallel data”, it refers to the sample data formed by taking multiple cross-sections from a time series and simultaneously selecting sample observations from these cross-sections. The data sample of this study is a certain data index of M objects (provincial administrative region) on N time nodes (years)) collected from 31 provincial administrative regions in China. Labor input and water consumption are set as input indicators, while GDP and wastewater are the output indicators to analyze wastewater efficiency and economic efficiency in the first stage of each province. Water pollutant COD is a link indicator, wastewater treatment expense is an input indicator, and wastewater treatment capacity and number of water diseases are output indicators in the second stage. The carry-over variable is fixed assets to help evaluate the efficiency of government wastewater input in each province. On the basis of the dynamic network DEA model proposed by Tone and Tsutsui [38], we add the undesirable output variables to form the modified undesirable dynamic network model.

#### Modified Undesirable Dynamic Network Model

Suppose there are *n DMU*s (*j* = 1, …, *n*), with each one having *k* divisions (*k* = 1, …, *K*) and *T* time periods (*t* = 1, …, *T*). Each *DMU* has an input and output at time period *t* and a carry-over (link) to the next *t* + 1 time period. We set $m_{k}$ and $r_{k}$ as the corresponding input and output in each division K, with (k,h)i representing divisions k to h, and $L_{hk}^{}$ is the k and h division set. The input and output, links, and carry-over definitions are outlined in the following.

Inputs and outputs

$X_{ijk}^{t}\in R_{+}\left(i=1,\ldots ,m_{k};⥂j=1,\ldots ,n;K=1\ldots ,K;t=1,\ldots ,T\right)$: refers to input i at time period t for $DMU_{j}$ division k.

$y_{rjk}^{t}\in R_{+}\left(r=1,\ldots ,r_{k};⥂j=1,\ldots ,n;K=1\ldots ,K;t=1,\ldots ,T\right)$: refers to output *r* in time period *t* for $DMU_{j}$ division k; if part of the output is not ideal, then it is considered an input for the division.

Links

$Z_{j(kh)t}^{t}\in R_{+}^{}$$(j=1;\ldots ;n;l=1;\ldots ;L_{hk}^{};t=1;\ldots ;T)0$: refers to the period *t* links from $DMU_{j}$ division k to division h, with $L_{hk}^{}$ being the number of k to h links, and Z^t^_j(kh)t_ ∈R_+_(*j* = 1, …, *n*; *l* = 1, …, *L_kh_*; *t* = 1, …, *T*).

Carry-overs

$Z_{jkl}^{\left(t,t+1\right)}\in R_{+}^{}$$\left(j=1,\ldots ,n;l=1,\ldots ,L_{k}^{};k=1,\ldots k,t=1,\ldots ,T-1\right)$: refers to the carry-over of t to the t+1 period from $DMU_{j}$ division k to division h, with $L_{k}^{}$ being the number of carry-over items in division k.

The following is the non-oriented model.

(a) Objective function
  The Overall efficiency $\left(\theta_{0}^{*}\right)$ can be calculated by equal (1).
  (1) $$\theta_{0}^{*}=\min\frac{\overset{T}{\underset{t=1}{\sum }}W^{t}\left[\overset{K}{\underset{k=1}{\sum }}W^{k}\left[1-\frac{1}{m_{k}+linkin_{k}+ninput_{k}}\left(\overset{m_{k}}{\underset{i=1}{\sum }}\frac{S_{iok}^{t-}}{x_{iok}^{t}}+\overset{linkin_{k}}{\underset{{\left(kh\right)}_{l}=1}{\sum }}\frac{s_{o{\left(kh\right)}_{l}in}^{t}}{z_{o{\left(kh\right)}_{l}in}^{t}}+\overset{ninput_{k}}{\underset{k_{l}}{\sum }}\frac{s_{ok_{l}input}^{\left(t,t+1\right)}}{z_{ok_{l}input}^{\left(t,t+1\right)}}\right)\right]\right]}{\overset{T}{\underset{t=1}{\sum }}W^{t}\left[\overset{K}{\underset{k=1}{\sum }}W^{k}\left[1+\frac{1}{r_{1k}+r_{2k}}\left(\overset{r_{1k}}{\underset{r=1}{\sum }}\frac{s_{rokgood}^{t+}}{y_{rokgood}^{t}}+\;\overset{r_{2k}}{\underset{r=1}{\sum }}\frac{s_{rokbad}^{t-}}{y_{rokbad}^{t}}\right)\right]\right]}$$
  Subject to:
  $$x_{ok}^{t}=X_{k}^{t}\lambda_{k}^{t}+s_{ko}^{t}\;\left(\forall k,\forall t\right)$$
  $$y_{okgood}^{t}=Y_{kgood}^{t}\lambda_{k}^{t}-s_{kogood}^{t+}\;\left(\forall k,\forall t\right)$$
  $$y_{okbad}^{t}=Y_{kbad}^{t}\lambda_{k}^{t}+s_{kobad}^{t-}\;\left(\forall k,\forall t\right)$$
  $$e\lambda_{k}^{t}=1\;\left(\forall k,\forall t\right)$$
  $$\lambda_{k}^{t}\geq 0,\;s_{ko}^{t-}\geq 0,s_{kogood}^{t+}\geq 0,s_{kobad}^{t-}\geq 0,\;\left(\forall k,\forall t\right)$$
  $$Z_{\left(kh\right)free}^{t}\lambda_{h}^{t}=Z_{\left(kh\right)free}^{t}\lambda_{k}^{t}\left(\forall \left(k,h\right)free,\forall t\right)$$
  $$Z_{\left(kh\right)free}^{t}=(Z_{1\left(kh\right)free}^{t},\ldots ,Z_{n\left(kh\right)free}^{t}\lambda_{k}^{t}\in R^{L_{\left(h\right)free}\times n}$$
  $$\;Z_{o\left(kh\right)fix}^{t}=Z_{\left(kh\right)fix}^{t}\lambda_{k}^{t}\left(\forall \left(k,h\right)fix,\forall t\right)$$
  $$Z_{o\left(kh\right)in}^{t}=Z_{\left(kh\right)in}^{t}\lambda_{k}^{t}+S_{o\left(kh\right)in}^{t}\left(\left(kh\right)in=1,\ldots ,linkin_{k}\right)$$
  $$\sum_{j=1}^{n}Z_{jk_{1}\alpha }^{\left(t,t+1\right)}\lambda_{jk}^{t}=\sum_{j=1}^{n}Z_{jk_{1}\alpha }^{\left(t,t+1\right)}\lambda_{jk}^{t+1}\left(\forall k;\forall k_{l};t=1,\ldots ,T-1\right)$$
  $$\;Z_{ok_{l}input}^{\left(t,t+1\right)}=\sum_{j=1}^{n}Z_{jk_{l}input}^{\left(t,t+1\right)}\lambda_{jk}^{t}-s_{ok_{l}input}^{\left(t,t+1\right)}k_{l}=1,\ldots ,ngood_{k};\forall k;\forall t)$$
  $$\;Z_{ok_{l}free}^{\left(t,t+1\right)}=\sum_{j=1}^{n}Z_{jk_{l}free}^{\left(t,t+1\right)}\lambda_{jk}^{t}-s_{ok_{l}free}^{\left(t,t+1\right)}k_{l}=1,\ldots ,nfree_{k};\forall k;\forall t)$$
  $$\;Z_{ok_{l}fix}^{\left(t,t+1\right)}=\sum_{j=1}^{n}Z_{jk_{l}fix}^{\left(t,t+1\right)}\lambda_{jk}^{t}-s_{ok_{l}fix}^{\left(t,t+1\right)}k_{l}=1,\ldots ,nfix_{k};\forall k;\forall t)$$
  $$\;s_{ok_{l}good}^{\left(t,\left(t+1\right)\right)}\geq 0,\;s_{ok_{l}free}^{\left(t,\left(t+1\right)\right)}:free\left(\forall k_{l};\forall t\right)$$
(b) Period and division efficiencies
  Period and division efficiencies are as follows:
  (b1) The Period efficiency $\partial_{0}^{*}$ can be calculated by equal (2).
    (2) $$\partial_{0}^{*}=\min\frac{\overset{K}{\underset{k=1}{\sum }}W^{k}\left[1-\frac{1}{m_{k}+linkin_{k}}(\overset{m_{k}}{\underset{i=1}{\sum }}\frac{S_{iok}^{t-}}{x_{iok}^{t}}+\overset{linkin_{k}}{\underset{{\left(kh\right)}_{l}=1}{\sum }}\frac{s_{o{\left(kh\right)}_{l}in}^{t}}{z_{o{\left(kh\right)}_{l}in}^{t}})\right]}{\overset{K}{\underset{k=1}{\sum }}W^{k}\left[1+\frac{1}{r_{1k}+r_{2k}+ngood_{k}}\left(\overset{r_{1k}}{\underset{r=1}{\sum }}\frac{s_{rokgood}^{t+}}{y_{rokgood}^{t}}+\;\overset{r_{2k}}{\underset{r=1}{\sum }}\frac{s_{rokbad}^{t-}}{y_{rokbad}^{t}}+\overset{ngood_{k}}{\underset{k_{l}}{\sum }}\frac{s_{ok_{l}good}^{\left(t,t+1\right)}}{z_{ok_{l}good}^{\left(t,t+1\right)}}\right)\right]}$$
  (b2) The Division efficiency $\varphi_{0}^{*}$ can be calculated by equal (3).
    (3) $$\varphi_{0}^{*}=\min\frac{\overset{T}{\underset{t=1}{\sum }}W^{t}\left[1-\frac{1}{m_{k}+linkin_{k}+ninput_{k}}\left(\overset{m_{k}}{\underset{i=1}{\sum }}\frac{S_{iok}^{t-}}{x_{iok}^{t}}+\overset{linkin_{k}}{\underset{{\left(kh\right)}_{l}=1}{\sum }}\frac{s_{o{\left(kh\right)}_{l}in}^{t}}{z_{o{\left(kh\right)}_{l}in}^{t}}+\overset{ninput_{k}}{\underset{k_{l}}{\sum }}\frac{s_{ok_{l}input}^{\left(t,t+1\right)}}{z_{ok_{l}input}^{\left(t,t+1\right)}}\right)\right]}{\overset{T}{\underset{t=1}{\sum }}W^{t}\;\left[1+\frac{1}{r_{1k}+r_{2k}}\left(\overset{r_{1k}}{\underset{r=1}{\sum }}\frac{s_{rokgood}^{t+}}{y_{rokgood}^{t}}+\;\overset{r_{2k}}{\underset{r=1}{\sum }}\frac{s_{rokbad}^{t-}}{y_{rokbad}^{t}}\right)\right]}$$
  (b3) The Division period efficiency $\rho_{0}^{*}$ can be calculated by equal (4).
    (4) $$\rho_{0}^{*}=\min\frac{1-\frac{1}{m_{k}+linkin_{k}+ninput_{k}}(\overset{m_{k}}{\underset{i=1}{\sum }}\frac{S_{iok}^{t-}}{x_{iok}^{t}}+\overset{linkin_{k}}{\underset{{\left(kh\right)}_{l}=1}{\sum }}\frac{s_{o{\left(kh\right)}_{l}in}^{t}}{z_{o{\left(kh\right)}_{l}in}^{t}}\overset{ninput_{k}}{\underset{k_{l}}{\sum }}\frac{s_{ok_{linput}input}^{\left(t,t+1\right)}}{z_{ok_{l}input}^{\left(t,t+1\right)}})}{1+\frac{1}{r_{1k}+r_{2k}}\left(\overset{r_{1k}}{\underset{r=1}{\sum }}\frac{s_{rokgood}^{t+}}{y_{rokgood}^{t}}+\;\overset{r_{2k}}{\underset{r=1}{\sum }}\frac{s_{rokbad}^{t-}}{y_{rokbad}^{t}}\right)}$$

From the above, we are able to obtain overall efficiency (1), period efficiency (2), division efficiency (3), and division period efficiency (4).

### 2.3. Labor, Water Consumption, Wastewater Treatment Expense, GDP, Wastewater Treatment Capacity, Wastewater, COD, and Water Diseases

There are eight key features of this present study: labor efficiency, water consumption efficiency, wastewater treatment expense efficiency, GDP efficiency, wastewater treatment capacity efficiency, wastewater efficiency, COD efficiency, and water diseases. In our study, “i” represents area, and “t” represents time. The eight efficiency models are defined in the following.

(5) $$\mathrm{Labor}\;\mathrm{efficiency}\;=\;\frac{\mathrm{Target}\;\mathrm{labor}\;\mathrm{input}\;\left(i,\;t\right)}{\mathrm{Actual}\;\mathrm{labor}\;\mathrm{input}\;\left(i,\;t\right)}$$

(6) $$\mathrm{Water}\;\mathrm{consumption}\;\mathrm{efficiency}\;=\;\frac{\mathrm{Target}\;\mathrm{water}\;\mathrm{input}\;\left(i,\;t\right)}{\mathrm{Actual}\;\mathrm{water}\;\mathrm{input}\;\left(i,\;t\right)}$$

(7) $$\mathrm{Wastewater}\;\mathrm{treatment}\;\mathrm{expense}\;\mathrm{efficiency}=\;\frac{\mathrm{Target}\;\mathrm{expense}\;\mathrm{input}\;\left(i,\;t\right)}{\mathrm{Actual}\;\mathrm{expense}\;\mathrm{input}\;\left(i,\;t\right)}$$

(8) $$\mathrm{GDP}\;\mathrm{efficiency}\;=\;\frac{\mathrm{Actual}\;\mathrm{GDP}\;\mathrm{desirable}\;\mathrm{output}\;\left(i,\;t\right)}{\mathrm{Target}\;\mathrm{GDP}\;\mathrm{desirable}\;\mathrm{output}\;\left(i,\;t\right)}$$

(9) $$\mathrm{Wastewater}\;\mathrm{treatment}\;\mathrm{capacity}\;\mathrm{efficiency}=\;\frac{\mathrm{Actual}\;\mathrm{capacity}\;\mathrm{desirable}\;\mathrm{output}\;\left(i,\;t\right)}{\mathrm{Target}\;\mathrm{capacity}\;\mathrm{desirable}\;\mathrm{output}\;\left(i,\;t\right)}$$

(10) $$\mathrm{Wastewater}\;\mathrm{efficiency}\;=\;\frac{\mathrm{Target}\;\mathrm{Wastewater}\;\mathrm{Undesirable}\;\mathrm{output}\;\left(i,\;t\right)}{\mathrm{Actual}\;\mathrm{Wastewater}\;\mathrm{Undesirable}\;\mathrm{output}\;\left(i,\;t\right)}$$

(11) $$\mathrm{COD}\;\mathrm{efficiency}\;=\;\frac{\mathrm{Target}\;\mathrm{COD}\;\mathrm{Undesirable}\;\mathrm{output}\;\left(i,\;t\right)}{\mathrm{Actual}\;\mathrm{COD}\;\mathrm{Undesirable}\;\mathrm{output}\;\left(i,\;t\right)}$$

(12) $$\mathrm{Water}\;\mathrm{diseases}\;=\;\frac{\mathrm{Target}\;\mathrm{diseases}\;\mathrm{Undesirable}\;\mathrm{output}\;\left(i,\;t\right)}{\mathrm{Actual}\;\mathrm{diseases}\;\mathrm{Undesirable}\;\mathrm{output}\;\left(i,\;t\right)}$$

In the above model, if the numerator and denominator are equal, then the calculated efficiency value is 1, which means the efficiency is optimal. If the numerator is less than the denominator, then the calculated efficiency value is greater than zero and less than 1, and smaller values mean less efficiency. Since the constraint condition has been set in the model, so that no numerator is greater than the denominator, the calculated efficiency value is at most 1.

## 3. Empirical Study

### 3.1. Data Sources and Description

**Figure 1:**
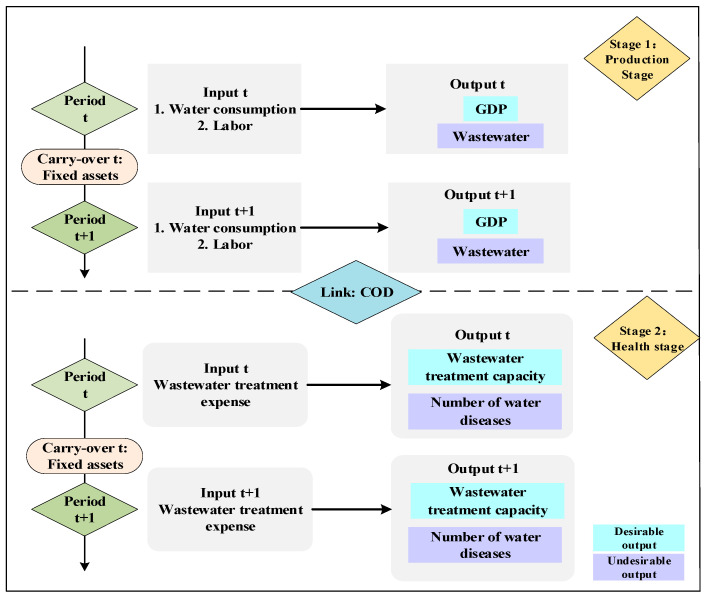
The modified undesirable dynamic network model.

This paper collects data of 31 provincial administrative regions in China from 2013 to 2017. The division of the eastern, central, western, and northeastern regions refers to the regional division standards published on the website of the National Bureau of Statistics of China. The eastern region includes Beijing, Tianjin, Hebei, Shanghai, Jiangsu, Zhejiang, Fujian, Shandong, Guangdong, and Hainan (10 provinces (cities)); the central region includes Shanxi, Anhui, Jiangxi, Henan, Hubei, and Hunan (6 provinces); the western region includes Inner Mongolia, Guangxi, Chongqing, Sichuan, Guizhou, Yunnan, Shaanxi, Gansu, Qinghai, Ningxia, Xinjiang, and Tibet (12 provinces or municipalities or autonomous regions); and the northeast region includes Liaoning, Jilin, and Heilongjiang (3 provinces). The data were extracted from the Statistical Yearbook of China, the Demographics and Employment Statistical Yearbook of China, the Environmental Yearbook of China, and the Health Statistics Yearbook of China. Figure 1 reveals the framework of the dynamic network model of the inter-temporal efficiency measurement and variables.

Table 1 shows all the input and output variables of the two stages. There are three inputs, four outputs, one link variable, and one carry-over variable.

Figure 1 shows the frame of the modified undesirable dynamic network model.

**Stage 1: Production Stage**

Input variables:
  Labor: This study takes the numbers of employees in each region by the end of each year. Unit: 10,000 persons.
  Water consumption: Gross amount of water taken by various water users, including loss of water delivery. Unit: 100 million tons.
  Fixed assets: The total amount of work done by the whole society in building and purchasing fixed assets and related expenses. Unit: 100 million RMB.
Output variables:
  Desirable output (GDP): Refers to the final result of production activities of all resident units in a region calculated by market price in a year. Unit: 100 million RMB.
  Undesirable output (Wastewater): It is the sum of industrial wastewater discharge and domestic sewage discharge. Unit: 10,000 tons.
Link between production stage and health stage variables:
  COD: The sum of chemical oxygen demand (COD) emissions from industrial wastewater and domestic wastewater. It refers to the amount of oxygen required to oxidize organic pollutants in water analyzed by chemical oxidizers.

**Stage 2: Health Stage**

Input variable:
  Wastewater treatment expense: The annual investment amount of each district’s wastewater treatment project. Unit: 10,000 RMB.
Output variables:
  Desirable output (wastewater treatment capacity): The amount of wastewater actually treated by various water treatment facilities. Unit: 10,000 tons.
  Undesirable output (number of water diseases): The number of water diseases caused by drinking polluted water and mainly includes fluorosis and arsenic poisoning. Water fluorosis and arsenic poisoning are two typical water poisoning diseases in China [39]. Unlike water diseases caused by common bacterial infections, they are chronic and regionally widespread. Unit: persons.

### 3.2. Statistical Analysis of Input-Output Indicators

Table 2 shows the statistical analysis for the original data of labor, water consumption, wastewater production, wastewater treatment expense, COD, and number of water diseases. From 2013 to 2017, the maximum and minimum values of labor input increased slowly, and the average value decreased slightly. This is mainly due to the disappearance of China’s demographic dividend and the slowdown of its population growth. At the same time, the number of the working-age population gradually declined.

The average and maximum values of wastewater discharge fluctuated distinctly. After peaking in 2015 (237,200.839), the average declined again in 2016–2017. The maximum value continued to rise over 2013–2016 from 862,471.080 to 938,261.030, and after peaking in 2016 there was a significant decline in 2017. The standard deviation also showed a trend of rising first and then falling, which indicates that regional differences are narrowing.

The maximum input of wastewater treatment expense has decreased significantly from 175,141 to 105,626 since 2014. The trend of average decline is also quite obvious. It shows that the investment cost of wastewater treatment in various provinces and municipalities in China has been decreasing year by year.

As the most important indicator of water pollution, COD has declined significantly after 2015 from 2.9 to 2.5, which means that the China government’s promulgation and implementation of the new “Environmental Protection Law” and “Water Pollution Prevention and Control Action Plan” in 2015 had remarkable results. However, it is noteworthy that the maximum COD in 2017 rebounded to an upward trend compared with 2016, which means that water pollution in individual provinces and cities became aggravated again.

The average number of water diseases showed a slow downward trend, but the maximum value decreased significantly with a decrease of 513,132 between 2016 and 2017, denoting that there are obvious regional differences in water pollution diseases. The areas with high incidences of water pollution diseases need more careful control, and the situation of prevention and control of water pollution diseases in China is still serious.

### 3.3. Analysis of the Total Efficiency of the Provinces from 2013 to 2017

**Figure 2:**
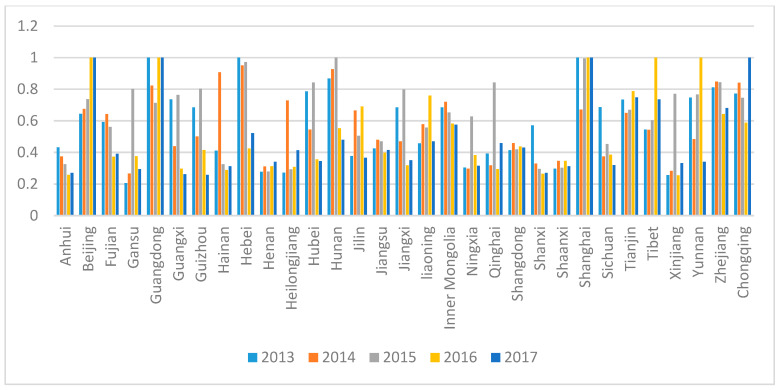
Total efficiency scores of provinces, municipalities, and autonomous regions from 2013 to 2017.

Figure 2 shows that the total efficiency scores of provinces, municipalities, and autonomous regions fluctuate greatly from 2013 to 2017. The provinces, municipalities, and autonomous regions with a total efficiency score of 1 in 2017 include Beijing, Guangdong, Shanghai, and Chongqing. Chongqing is in the western region, while the others are in the eastern region. Some provinces, municipalities, and autonomous regions presented a steep increase in 2016, whose total efficiency score increased significantly, such as compared with the previous year, Beijing increased by 35.7% and Guangdong by 40.15%. In 2014, the average score of total efficiency in northeast China rose from 0.3603 in 2013 to 0.6576. In 2015, some provinces increased significantly. For example, compared with 2014, Gansu increased by 201.47% and Qinghai by 164.55%. However, these provinces generally fell sharply in the year after their sharp rise, which led to a downward trend in the eastern, central, and western regions, except for the average level of total efficiency in the northeast region. Although the average score of total efficiency in the eastern region decreased from 0.7031 to 0.6502, it remains the best among the four regions.

From the total efficiency ranking, the ranking of most cities fluctuates greatly, and the provinces with higher ranking are Beijing, Shanghai, Jiangsu, Shandong, and Tianjin in the eastern region. With great variation, the northeastern region has been on the rise. From this, we can see that the rankings of most provinces and cities in the central and eastern regions declined, while the rankings of provinces and cities in the northeast and western regions increased significantly.

Table 3 shows that the provinces, municipalities, and autonomous regions with a total efficiency of 1 for five consecutive years are Beijing, Guangdong, Inner Mongolia, Shanghai, Tianjin, and Tibet. In the eastern, western, and northeastern regions, the average level of total efficiency scores in the first stage has been rising. In Stage 1, the total efficiency scores of most cities in China also showed an upward trend, with Chongqing and Qinghai presenting the greatest increase. The average level of the total efficiency score in Stage 1 of the eastern region is relatively stable and the best among the four regions. The average water fluctuation of the first stage efficiency score in the central region is not significant, but overall has declined. The average levels of Stage 1 in the western and northeastern regions have made remarkable progress.

The total efficiency score of Stage 2 is quite different from that of Stage 1. For example, in 2017 the efficiency score of Tianjin in Stage 1 was 1, and that in Stage 2 was only 0.4976. In 2013, their total efficiency scores in Stage 2 were all DEA-effective, and in 2017 these provinces all dropped to below 0.5.

**Figure 3:**
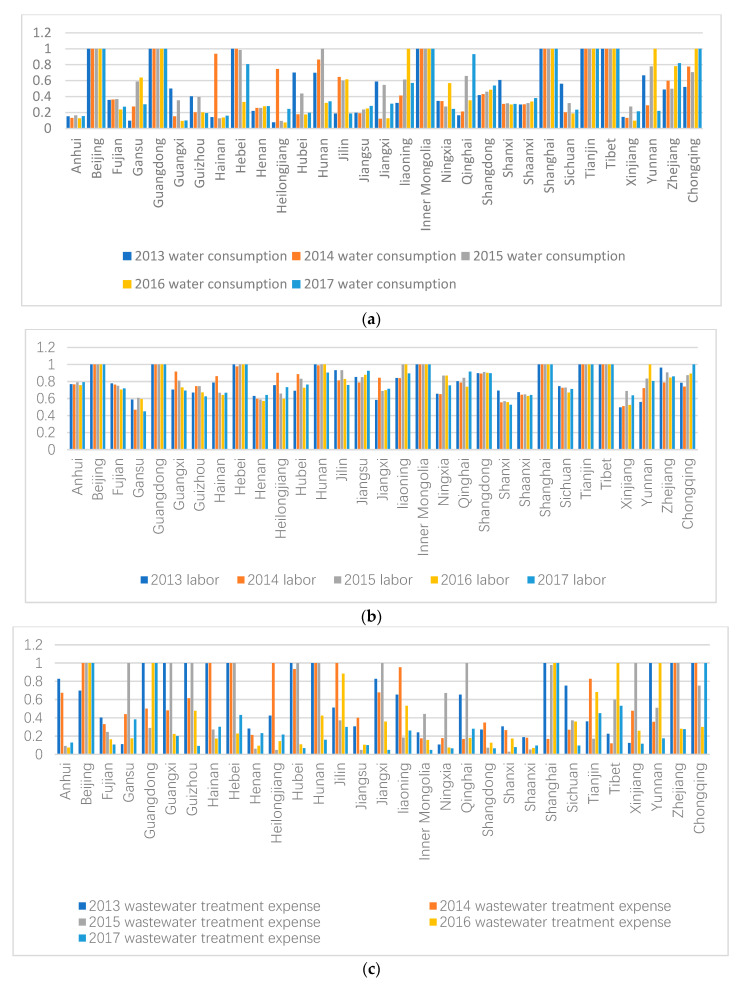
(**a**) Water consumption efficiency in provinces, municipalities, and autonomous regions from 2013 to 2017. (**b**) Labor efficiency of provinces, municipalities, and autonomous regions from 2013 to 2017. (**c**) Wastewater treatment cost and efficiency in provinces, municipalities, and autonomous regions from 2013 to 2017.

Figure 3a–c reflect the efficiency changes of water consumption, labor force, and wastewater treatment cost.

Water consumption efficiency has fluctuated with a decrease in Fujian, Guizhou, Hainan, Hebei, Hubei, Hunan, Jiangxi, Ningxia, Shanxi, Sichuan, Xinjiang, and Guangxi. From 2013 to 2017, the water consumption efficiency in the eastern region maintained a stable level, with no significant increase. In the past five years, water consumption efficiency in the central region has gradually decreased, rising only in 2017, but still at the low efficiency level of 0.265. Water consumption efficiency in the western region fluctuated from 0.4755 in 2013 to 0.4075 in 2014 to 0.5555 in 2015, but decreased year by year after 2015 to 0.4855 in 2017. Water consumption efficiency of the northeastern region is the same as that of west China, but the efficiency of water consumption in the northeastern region increased greatly in 2014, from 0.195 in 2013 to 0.602 in 2014.

From the perspective of labor efficiency, the eastern, central, western, and northeastern regions are in a stable state. However, labor efficiency in the eastern region is still higher than that in the other three regions at about 0.9, versus the central region at about 0.7, the western region at about 0.7, and the northeastern region at about 0.8. The efficiency of labor in different regions is similar, and the space for improvement is limited.

According to the input efficiency scores of wastewater treatment expense, the average level of the four regions has been declining, and the central region has the greatest decline. By 2017, the central region had become the region with the lowest input efficiency of wastewater treatment. Although the eastern region has declined, it is still the best of the four regions. The situation in the northeast is similar to that in the west. In the eastern region, Beijing, Shanghai, Guangdong, Zhejiang, and Hebei provinces maintain three years or more of optimal efficiency, but the overall efficiency of wastewater treatment expenses of Jiangsu is less efficient than most eastern provinces. Most cities have dropped to a lower level.

In the eastern region, only Beijing and Shanghai have maintained DEA validity in the past three years. The scores of other non-DEA-effective regions have fluctuated greatly in five years with a big gap between them. The province with the greatest decline is Hubei, whose input efficiency score of wastewater treatment cost was 1 in 2013 and 0.0658 in 2017.

Table 4 lists the average efficiency values of wastewater and COD in four regions from 2013 to 2017. The wastewater discharge efficiency of the eastern region is better than that of the other three regions, and the wastewater discharge efficiency of the central region is the lowest among the four regions.

The average COD efficiency scores on the whole in northeast China have risen the most and made the most obvious progress. However, the average level is still not high and fluctuated significantly. The average scores of COD efficiency in the eastern, central, and western regions have decreased, especially in the central region. In 2016, the average score of COD efficiency in the central region was only 0.3799. The average score in the eastern region fell slightly, but was still the best of the four regions, followed by the west, with the central region as the worst.

Table 4 also presents the output efficiency score of Stage 2’s water diseases and water treatment capacity in 2013–2017. The efficiencies of water diseases in all four regions are on the decline. The efficiency of the eastern region generally dropped and reached its lowest level in 2016, with an efficiency of only around 0.4. The efficiency of water pollution in the central region also generally fell. From 2013 to 2014, its efficiency declined the fastest, from 0.78 to 0.40, and then reached the lowest in 2017 at only 0.38. The western region rose from 0.46 to 0.88 between 2014 and 2015, which was the best efficiency among the four regions for the five years of statistics. In the northeastern region, the efficiency was basically stable in the first two years, but it fluctuated greatly in the next three years, from 0.34 in 2014 to 0.84 in 2015. However, in 2016 and 2017, the efficiency was only 0.03 and 0.06, which was greatly different from that before.

From the viewpoint of wastewater treatment efficiency, the eastern region has been in a stable state as a whole, with efficiency sustained at around 0.8. The efficiency of the central region in 2013 and 2014 was DEA-efficient, but there has been a slight decline since then. The western region as a whole is in a trending decline, from the original level of 0.85 in 2013 to 0.50 in 2017. The overall efficiency of the northeast region looks to be the best in all regions. The efficiencies of the first four years were DEA-efficient, but then fell to 0.74 in 2017.

**Figure 4:**
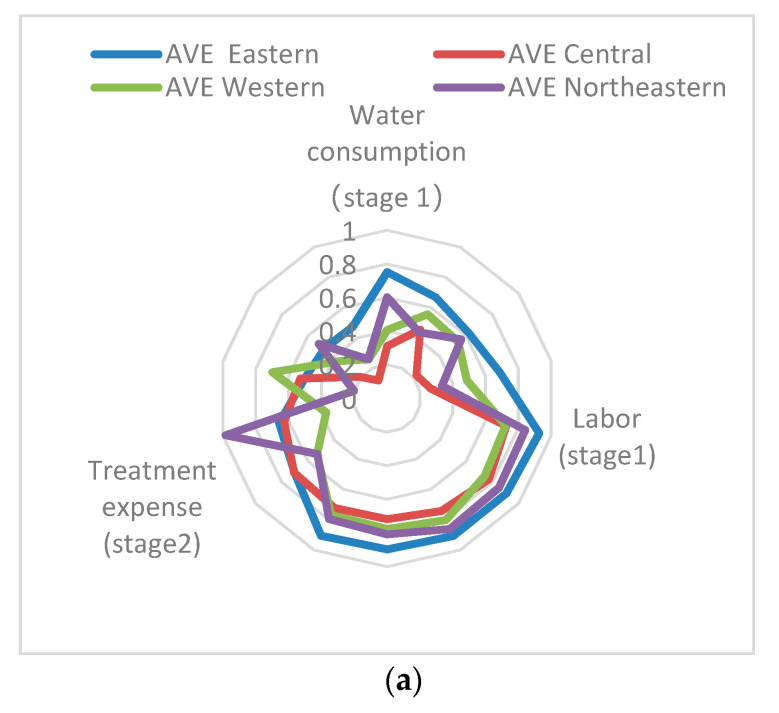

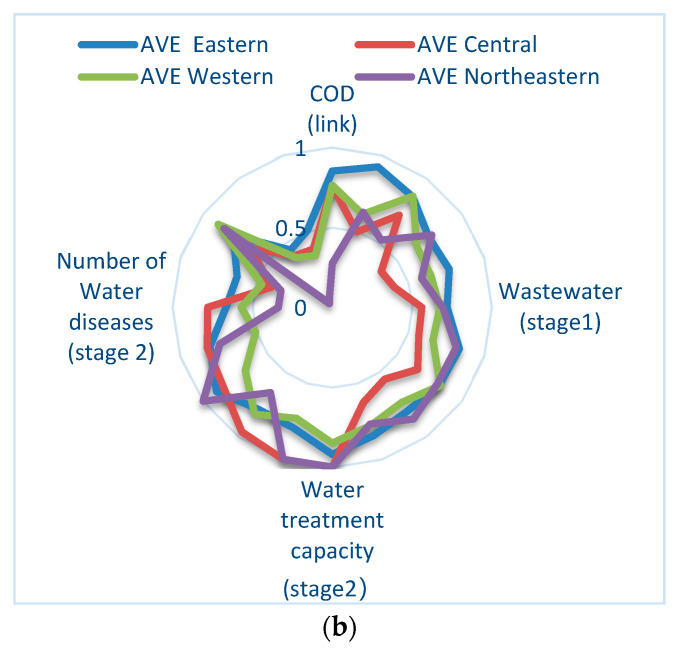
(**a**) Input variables radar map from 2013 to 2017. (**b**) Output variables’ radar map from 2013 to 2017.

Figure 4a,b show the input and output variables’ radar map from 2013 to 2017. From Figure 4a, the efficiency scores of the three input variables in the eastern region are better than those in the other three regions, and the differences in scores of the variables in the eastern region vary. However, the efficiency of input variables in the central, western, and northeastern regions is greatly unbalanced. Among the three input variables, the scores of labor efficiency are better than those of water consumption and treatment expense.

From Figure 4b, the efficiency score of water treatment capacity of the four output variables is generally better than the other three output variables, and the four regions are all at a higher efficiency level. From a regional perspective, there is strong correlation between the COD efficiency score and water diseases, and the situation of water diseases is relatively worse in areas with high COD efficiency. Except for water treatment capacity, the output variable efficiency in the eastern region is generally better than the other three regions. There is still room for improvement in the central and northeastern regions. All typical value of indicator can be found in Table 5.

**Figure 5:**
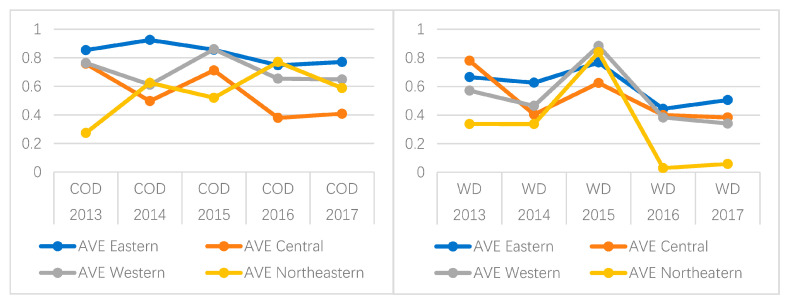
Chemical oxygen demand (COD) and water diseases (WD) line charts from 2013 to 2017.

As an important indicator of organic pollution in water bodies, COD can reflect the degree of water pollution, and so the comparative analysis of COD and water disease efficiency can be used as an important basis for the degree of association between wastewater pollution and water diseases. Figure 5 is drawn to analyze the specific values and changes of these two variables during 2013–2017. It is relatively clear and intuitive that the efficiency values of COD and water diseases show basically the same fluctuation trend. Taking the central region as an example, the efficiency of COD decreased from 0.7588 in 2013 to 0.4927 in 2014, and the efficiency of water diseases also decreased from 0.7802 in 2013 to 0.4043 in 2014. The efficiency of COD then increased from 0.4972 to 0.7124 from 2014 to 2015, correspondingly, and the efficiency of water diseases also increased from 0.4043 in 2014 to 0.6246 in 2015. The only exception is that the efficiency of COD and water disease in northeastern China is significantly different, and the fluctuation does not show a consistent trend. In 2016 and 2017, the efficiency of water disease control was obviously extremely low. The natural unfavorable conditions of high fluorine and arsenic content in underground diving, coupled with the realistic situation of industrial wastewater discharge by heavy industry, make the northeastern region one of the serious areas of local water diseases in China, and so it is urgent to prevent and control water pollution and water diseases. As a further subdivision and in-depth direction of this issue, the specific influencing factors of water pollution and water diseases in China can be explored through the collection and classification of more relevant data in the future.

The quality of treated water is closely related to the risk associated with water consumption due to the great difference in water quality in different regions of China. At present, the effluent from China’s wastewater treatment plants only reaches I-Type which is far lower than III-Type (centralized drinking water source) water quality on the surface. Therefore, the quality of treated water brings two risks. One risk is the lack of clean water as the water source of the waterworks, and the other is the health risk of residents due to substandard water quality. It is necessary to change the wastewater into III-Type or more standard surface water purification in the link of the final wastewater treatment plant so as to form a closed loop in the water cycle. The risks associated with water consumption can truly be addressed through the formation of clean water.

Table 6 shows the Kruskal–Wallis H test score for the average score of the four regions in China. In 2014, 2016, and 2017, the average total efficiencies all pass the significance test.

## 4. Discussion

Comparing our results with previous studies like Chen et al. [40], Wang and Yang [19], and Chen et al. [41], we find some similarities and differences.

(1) The severity of water pollution depends mainly on population density, types and quantities of industrial and agricultural development, and the number and efficiency of wastewater treatment systems. However, this study does not consider the influence of population density, even though Chen et al. [41] pointed out that the population scale effect has a positive contribution to wastewater discharge, but the impact is not significant.
(2) Water pollution shows a significant association with regional water diseases like arsenic poisoning and fluorosis. Compared with previous studies by Chen et al. [40] and Wang and Yang [19], this research further explores the regional differences between wastewater pollution and health. The efficiency of water disease prevention and control in all four regions of China shows a downward trend, and the severe situation of water disease control in the northeast indicates that China’s wastewater pollution and water disease control have not been optimistic in recent years, and thus measures are needed for improvement.
(3) The provinces with high wastewater discharge can set up industrial parks. In these parks, they can introduce advanced wastewater treatment like membrane technology (Zheng et al. [42]) to remove harmful substances in wastewater.

Due to the lag of data statistics, this study only updated the subdivision data of provincial administrative regions in China to 2017. In 2018, the total amount of wastewater discharged in China was 69,966.1 million tons, including 34,317 kg of arsenic [43]. In 2019, the quality of China’s surface water environment continued to improve. The water quality of the main rivers and lakes has risen, while poor water quality of V-type has fallen. Of the 902 monitoring sections (points) for domestic and drinking water sources in China, 830 reached the standard for the whole year, accounting for 92.0% [44]. The release of the latest figures shows that the China government is aware of the dangers of wastewater pollution and the importance of its treatment, despite the country’s high levels of effluent and hazardous substances. China’s water environment and quality are gradually improving.

The data should be further updated in a follow-up study to match the latest reality of wastewater treatment and residents’ health in China. At the same time, the number of research samples (such as at prefecture-level cities) can be expanded to better study the subtle differences among regions.

## 5. Conclusions

From the perspective of total efficiency indicators, we offer the following conclusions.

(1) In addition to the increase in the average efficiency level in the northeastern region, the eastern, western, and central regions showed a downward trend. The eastern region performed best overall.
(2) The cost–benefit of wastewater treatment investment in the four regions has declined, with the central region having the largest decline. The situation in northeast China is similar to that in west China, and the efficiency in most areas has further declined.
(3) Wastewater discharge efficiency in the central region is at the lowest level in the past five years. COD output fluctuates significantly, and although the efficiency of the eastern region has declined, the efficiency is still optimal. Since water efficiency in the central region is the lowest among the four regions, attention should be paid to improving relevant technical policies and standards to improve water consumption efficiency.
(4) The efficiency of wastewater treatment is basically stable, and the efficiency of wastewater treatment in northeast China is the best. However, the scores of the occurrence efficiency of water disasters in the northeast region is the worst.

Timely updated wastewater treatment systems and installations can also improve wastewater utilization efficiency, enhance wastewater reusability, encourage reuse of wastewater, and reduce direct and indirect discharges of wastewater. At the same time, the relevant authorities must pay attention to the safety of wastewater reuse and avoid unnecessary harm to public health.

(1) The efficiencies of prevention and control of water diseases in all four regions are declining. There is a close relationship between COD and water disease efficiency. In the central and western regions, there is a positive correlation between the two scores. However, the effect of COD efficiency on water health efficiency in the eastern and northeastern regions is limited. The eastern region should therefore pay more attention to the control of COD content and improve the requirements of corresponding indicators, so as to reduce the number of water pollution diseases and achieve the goal of improving the overall efficiency.

Due to the limited natural purification capacity of the water resources, the four regions at the same time should further adjust its industrial structure and reasonably build facilities for centralized treatment of wastewater. The respective governments can establish complete environmental management archives by thoroughly checking the pollution information of enterprises.

In summary, the overall situation in the eastern region is better than that in the central, western, and northeastern regions. China has a vast amount of territory, a clear regional gap, a large economic gap, and large differences in economic and social development.

## 6. Recommendations

In the aspect of COD reduction and prevention and control of water diseases, the following measures should be actively carried out.

(1) Strengthen water quality monitoring of upstream water sources and conduct regular water source pollution surveys. Due to the strong correlation between COD and water pollution, water quality testing and pollution control measures should be strengthened. Upstream monitoring can focus on and select projects that have an impact on water quality. The sensory properties of water such as turbidity and odor, organic matter pollution, eutrophication, and microbial indicators of bacterial contamination should be targeted. At the same time, according to the type of water pollution, a regular survey can be conducted. The water samples of sewage discharge ports must be entrusted to health and epidemic prevention or environmental protection departments for analysis, and the survey results can then be compiled into written materials to predict the trend of pollution development.
(2) Strengthen the classified collection and treatment of wastewater. According to the water quality characteristics of different wastewater classification collections and treatments, a process should be set up to reduce the cost of treatment and also ensure a better treatment effect. For example, the classification collection and separate treatment of industrial wastewater containing precious metals can recover precious metals in wastewater, effectively reduce the possibility of excessive heavy metals in industrial wastewater, and also create additional value for enterprises. At the same time, the government should establish a scientific charging mechanism for urban water and wastewater treatment and use pricing policies to jointly adjust the demand for drainage and to reduce the amount of sewage.
(3) Govern pollution sources according to law. The prevention and control of water pollution highly correlate with the health of residents, have far-reaching effects, and must be regulated and guaranteed through laws. Polluting entities that have affected the quality of water resources must be treated according to laws that rely on closely coordinated management among central and local governments, environmental protection, and health departments. At the same time, related organizations can strengthen media publicity and guidance, enhance public water source protection and wastewater reuse awareness, and pay attention to the health problems and their root causes brought about by wastewater discharge.

## Figures and Tables

**Table 1:** Input and output variables.

Process	Input Variable	Output Variable	Link	Carry-Over
Stage 1	Labor	GDP	COD	Fixed assets
	Water consumption	Wastewater		
Stage 2	Wastewater treatment expense	Wastewater treatment capacity Number of water diseases		

**Table 2:** Statistical analysis of input–output indicators from 2013 to 2017.

Stage 1	**Input Variable**	**Index**	**2013**	**2014**	**2015**	**2016**	**2017**	**Input** **Variable**	**Index**	**2013**	**2014**	**2015**	**2016**	**2017**
	Water consumption	Average	199.468	196.606	196.890	200.307	194.948	Labor	Average	584.144	589.606	582.661	577.035	569.156
		MIN	23.800	24.100	25.700	26.400	25.800		MIN	31.020	32.540	33.390	31.510	33.300
		MAX	588.000	591.300	577.200	577.400	591.300		MAX	1966.980	1973.280	1948.040	1957.570	1963.100
		St Dev	149.015	149.289	147.533	145.104	145.923		St Dev	430.829	439.135	431.505	428.209	426.971
	**Output Variable**		**2013**	**2014**	**2015**	**2016**	**2017**	**Link**		**2013**	**2014**	**2015**	**2016**	**2017**
	Wastewater	Average	224,336.539	231,024.211	237,200.839	229,385.609	225,697.096	COD	Average	75.887	74.019	71.726	33.755	32.971
		MIN	5004.680	5449.680	5883.000	6142.750	7175.650		MIN	2.600	2.800	2.900	2.700	2.500
		MAX	862,471.080	905,082.060	911,523.000	938,261.030	882,020.480		MAX	184.600	178.000	175.800	96.400	100.100
		St Dev	184,430.104	190,473.093	195,601.802	194,225.740	185,112.232		St Dev	49.657	48.145	46.941	22.542	23.151
Stage 2	**Input variable**		**2013**	**2014**	**2015**	**2016**	**2017**	**Output variable**		**2013**	**2014**	**2015**	**2016**	**2017**
	Treatment expense	Average	40,284.613	37,176.581	38,198.000	34,916.000	24,637.387	Number of water diseases	Average	639,241.806	481,404.129	498,569.323	515,184.581	478,087.710
		MIN	572.000	90.000	893.000	15.000	280.000		MIN	673.000	673.000	49.000	881.000	1394.000
		MAX	15,0634.000	175,141.000	164,862.000	158,518.000	105,626.000		MAX	4,894,181.000	4,960,904.000	4,908,054.000	2,710,593.000	2,197,461.000
		St Dev	38,242.050	37,554.249	41,606.657	38,104.037	28,491.368		St Dev	1,033,514.768	1,002,811.200	1,022,375.107	738,905.138	690,593.449

**Table 3:** Total efficiency scores and ranks of provinces, municipalities, and autonomous regions from 2013 to 2017.

DMU		2013 (1)	2013 (2)	2013 Score	Rank	2014 (1)	2014 (2)	2014 Score	Rank	2015 (1)	2015 (2)	2015 Score	Rank	2016 (1)	2016 (2)	2016 Score	Rank	2017 (1)	2017 (2)	2017 Score	Rank	AVE
																						2013–2017
**Eastern region**	Beijing	1	0.2871	0.6436	15	1	0.3494	0.6747	9	1	0.4739	0.7369	14	1	1	1	1	1	1	1	1	
	Fujian	0.4835	0.7017	0.5926	16	0.6203	0.6646	0.6425	13	0.626	0.4966	0.5613	20	0.5186	0.2285	0.3735	20	0.545	0.2381	0.3916	16	
	Guangdong	1	1	1	1	1	0.6453	0.8226	6	1	0.427	0.7135	15	1	1	1	1	1	1	1	1	
	Hainan	0.3612	0.4612	0.4112	23	0.815	1	0.9075	3	0.4201	0.231	0.3256	26	0.4097	0.1696	0.2896	28	0.4213	0.2046	0.3129	25	
	Hebei	1	1	1	1	0.9029	1	0.9514	1	0.9451	1	0.9725	3	0.5861	0.2642	0.4252	14	0.8697	0.1747	0.5222	9	
	Jiangsu	0.4391	0.4095	0.4243	21	0.5582	0.4002	0.4792	19	0.6008	0.3401	0.4705	23	0.616	0.1791	0.3975	16	0.669	0.1608	0.4149	14	
	Shandong	0.5594	0.2688	0.4141	22	0.6449	0.2716	0.4583	21	0.653	0.1855	0.4192	25	0.6686	0.2058	0.4372	13	0.6878	0.174	0.4309	13	
	Shanghai	1	1	1	1	1	0.3407	0.6704	10	1	0.9894	0.9947	2	1	1	1	1	1	1	1	1	
	Tianjin	1	0.4678	0.7339	10	1	0.301	0.6505	12	1	0.3384	0.6692	16	1	0.5766	0.7883	6	1	0.4976	0.7488	5	
	Zhejiang	0.6236	1	0.8118	5	0.6964	1	0.8482	4	0.6865	1	0.8433	4	0.7959	0.4886	0.6423	9	0.7943	0.5673	0.6808	7	
	AVE	0.7467	0.6596	0.7031		0.8238	0.5973	0.7105		0.7931	0.5482	0.6707		0.7595	0.5112	0.6354		0.7987	0.5017	0.6502		0.674
**Central region**	Anhui	0.3566	0.506	0.4313	20	0.4067	0.344	0.3753	24	0.4328	0.2174	0.3251	27	0.4151	0.1005	0.2578	30	0.4393	0.1035	0.2714	29	
	Henan	0.3338	0.2216	0.2777	28	0.4306	0.1919	0.3112	28	0.4255	0.1337	0.2796	31	0.427	0.1987	0.3128	24	0.4396	0.2423	0.341	21	
	Hubei	0.5717	1	0.7858	6	0.5308	0.5579	0.5443	15	0.6856	1	0.8428	6	0.4783	0.2356	0.357	21	0.4936	0.1984	0.346	19	
	Hunan	0.7351	1	0.8676	4	0.8538	1	0.9269	2	1	1	1	1	0.6595	0.4462	0.5529	12	0.6348	0.3246	0.4797	10	
	Jiangxi	0.457	0.9139	0.6854	12	0.461	0.4775	0.4693	20	0.5983	1	0.7992	9	0.4067	0.2295	0.3181	23	0.5228	0.1781	0.3504	18	
	Shanxi	0.5884	0.5541	0.5712	17	0.4178	0.2427	0.3302	26	0.4215	0.1715	0.2965	29	0.4008	0.13	0.2654	29	0.4341	0.1088	0.2715	28	
	AVE	0.5071	0.6993	0.6032		0.5168	0.4690	0.4929		0.5939	0.5871	0.5905		0.4646	0.2234	0.3440		0.4940	0.1926	0.3433		0.4748
**Western region**	Inner Mongolia	1	0.3692	0.6846	14	1	0.4396	0.7198	8	1	0.3049	0.6525	17	1	0.1657	0.5828	11	1	0.1498	0.5749	8	
	Xinjiang	0.2596	0.2553	0.2574	30	0.421	0.1463	0.2836	30	0.5404	1	0.7702	10	0.3695	0.1401	0.2548	31	0.5409	0.1254	0.3331	22	
	Yunnan	0.4943	1	0.7472	8	0.5056	0.4632	0.4844	18	0.7778	0.7551	0.7664	11	1	1	1	1	0.4807	0.2024	0.3416	20	
	Ningxia	0.3878	0.2204	0.3041	26	0.4678	0.1283	0.298	29	0.63	0.6245	0.6273	18	0.6679	0.0995	0.3837	18	0.546	0.0856	0.3158	24	
	Qinghai	0.3906	0.3958	0.3932	24	0.4461	0.1909	0.3185	27	0.6857	1	0.8429	5	0.4827	0.1072	0.295	27	0.8024	0.1158	0.4591	12	
	Shaanxi	0.4258	0.1695	0.2977	27	0.4719	0.2215	0.3467	25	0.4573	0.1498	0.3035	28	0.4637	0.231	0.3473	22	0.4811	0.1444	0.3127	26	
	Sichuan	0.5857	0.7876	0.6866	11	0.4734	0.2778	0.3756	23	0.5496	0.3553	0.4524	24	0.4519	0.3197	0.3858	17	0.4739	0.1651	0.3195	23	
	Chongqing	0.5432	1	0.7716	7	0.6834	1	0.8417	5	0.732	0.7598	0.7459	13	0.7812	0.394	0.5876	10	1	1	1	1	
	Gansu	0.2745	0.1377	0.2061	31	0.402	0.1298	0.2659	31	0.6032	1	0.8016	8	0.6411	0.1106	0.3758	19	0.4391	0.1497	0.2944	27	
	Guangxi	0.471	1	0.7355	9	0.5032	0.3744	0.4388	22	0.6071	0.9217	0.7644	12	0.4169	0.1789	0.2979	26	0.3938	0.1312	0.2625	30	
	Guizhou	0.467	0.903	0.685	13	0.4743	0.5264	0.5004	17	0.6053	1	0.8027	7	0.4542	0.3746	0.4144	15	0.4168	0.0984	0.2576	31	
	Tibet	1	0.089786	0.544893	18	1	0.086604	0.543302	16	1	0.207483	0.603741	19	1	1	1	1	1	0.472495	0.736247	6	
	AVE	0.5351	0.5887	0.5619		0.5831	0.3902	0.4866		0.6990	0.7199	0.7095		0.6282	0.3426	0.4854		0.6237	0.2388	0.4313		0.5349
**North-eastern region**	Jilin	0.4652	0.2895	0.3773	25	0.7567	0.5744	0.6655	11	0.844	0.1657	0.5048	22	0.7493	0.6306	0.6899	8	0.4775	0.2549	0.3662	17	
	Heilongjiang	0.3295	0.2163	0.2729	29	0.8138	0.6443	0.7291	7	0.4963	0.0908	0.2935	30	0.4633	0.1537	0.3085	25	0.6595	0.1685	0.414	15	
	Liaoning	0.4981	0.4174	0.4578	19	0.5605	0.5957	0.5781	14	0.7302	0.3832	0.5567	21	1	0.5179	0.759	7	0.7021	0.2385	0.4703	11	
	AVE	0.4309	0.3078	0.3693		0.7103	0.6048	0.6576		0.6901	0.2132	0.4517		0.7375	0.4341	0.5858		0.6130	0.2206	0.4168		0.4962

**Table 4:** The average scores of input and output variables in Stage 1 and Stage 2 from 2013–2017.

Input	Year	AVE Eastern	AVE Central	AVE Western	AVE Northeastern	Output	Year	AVE Eastern	AVE Central	AVE Western	AVE Northeastern
Water consumption (stage)	2013	0.6602	0.4953	0.4755	0.1952	COD (link)	2013	0.8538	0.7588	0.7634	0.2740
	2014	0.7517	0.3102	0.4075	0.6017		2014	0.9249	0.4972	0.6117	0.6246
	2015	0.6675	0.4536	0.5555	0.4370		2015	0.8561	0.7124	0.8602	0.5203
	2016	0.6224	0.2219	0.5400	0.5637		2016	0.7475	0.3799	0.6535	0.7704
	2017	0.6878	0.2652	0.4855	0.3344		2017	0.7702	0.4088	0.6484	0.5885
	AVE	0.6779	0.3492	0.4928	0.4264		AVE	0.8305	0.5514	0.7074	0.5556
Labor (stage 1)	2013	0.9271	0.7264	0.7227	0.8420	Wastewater (stage 1)	2013	0.7192	0.5607	0.6742	0.6916
	2014	0.9063	0.7718	0.7415	0.8501		2014	0.8361	0.5696	0.6628	0.8165
	2015	0.9080	0.7431	0.8035	0.8620		2015	0.8286	0.662	0.8343	0.8147
	2016	0.8968	0.7174	0.7760	0.8078		2016	0.8021	0.5572	0.7374	0.8653
	2017	0.9064	0.7226	0.7687	0.7951		2017	0.8431	0.6272	0.7783	0.7684
	AVE	0.9089	0.7363	0.7625	0.8314		AVE	0.8058	0.5953	0.7374	0.7913
Treatment expense (stage 2)	2013	0.7041	0.7071	0.5337	0.5301	Water treatment capacity (stage 2)	2013	0.9214	1.0000	0.8511	1.0000
	2014	0.6574	0.6272	0.3719	0.9848		2014	0.7925	1.0000	0.7299	1.0000
	2015	0.5073	0.5302	0.6999	0.2018		2015	0.7888	0.9640	0.8281	0.6594
	2016	0.4759	0.2059	0.3560	0.5200		2016	0.8961	0.8444	0.6736	1.0000
	2017	0.4730	0.1193	0.2568	0.2593		2017	0.8063	0.8233	0.5043	0.7406
	AVE	0.5635	0.4379	0.4437	0.4992		AVE	0.8410	0.9263	0.7174	0.8800
The average scores						Number of water diseases (stage 2)	2013	0.6655	0.7802	0.5710	0.3381
							2014	0.6261	0.4043	0.4644	0.3378
							2015	0.7677	0.6246	0.8832	0.8390
							2016	0.4435	0.3995	0.3833	0.0296
							2017	0.5054	0.3834	0.3410	0.0584
							AVE	0.6016	0.5184	0.5286	0.3206

**Table 5:** The typical value of indicator

	Indicator	Water Consumption	Labor	Treatment Expense	COD	Wastewater	Water Diseases	Water Treatment Capacity
Region								
Ave. Eastern		Beijing (1)	Hainan (0.7240)	Jiangsu (0.1915)	Hainan (0.4276)	Hainan (0.5877)	Jiangsu (0.1708)	Beijing (0.5572)
Ave. Central		Hunan (0.6448)	Shanxi (0.5792)	Shanxi (0.1703)	Anhui (0.3465)	Henan (0.5312)	Hunan (0.9305)	Shanxi (0.7714)
Ave. Western		Inner Mongolia (1)	Gansu (0.5406)	Chongqing (0.8106)	Sichuan (0.4617)	Gansu (0.7982)	Ningxia (0.2351)	Tibet (0.4079)
Ave. Northeastern		Jilin (0.5835)	Liaoning (0.9139)	Heilongjiang (0.3669)	Heilongjiang (0.4603)	Jilin (0.8534)	Jilin (0.2269)	Liaoning (1)

**Table 6:** Kruskal–Wallis H Test Score of total efficiency.

Year	Ave. Northeastern	Ave. Eastern	Ave. Central	Ave. Western	Kruskal–Wallis Test Score
2013	0.3693	0.7031	0.6032	0.5619	0.138
2014	0.6576	0.7105	0.4929	0.4866	0.019 ***
2015	0.4517	0.6707	0.5905	0.7095	0.362
2016	0.5858	0.6354	0.3440	0.4854	0.087 **
2017	0.4168	0.6502	0.3433	0.4313	0.041 ***

Notes: *** significant confidence interval of 0.05 (two-tailed test). ** significant confidence interval of 0.1 (two-tailed test). The data in Table 5 are calculated with SPSS Statistics 25 Software (IBM Corporation., Armonk, New York, NY, USA).

## Appendix A

The China government has provided a quality standard for surface water (GB 3838-2002). China’s surface water falls into the following five categories.

**Table A1:** The quality standard for surface water.

Water Quality Classification	Water Quality	Characterization of Color	Function(s)
I-Type and II-Type water	Superior	Blue	Grade I pertains to source water and national nature reserves. Grade II includes first-class protected areas of centralized drinking water sources, precious fish protected areas, and fish and shrimp spawning grounds.
III-Type	Good	Green	Grade III relates to second-class protected areas of centralized drinking water sources and general fish protection and swimming zones.
IV-Type	Mild pollution	Yellow	Grade IV pertains to general industrial and recreational water areas where the human body is in direct contact with.
V-Type	Moderate pollution	Orange	Grade V refers to agricultural water use areas and waters with general landscape requirements.
Inferior V-type	Severe pollution	Red	The function of inferior V-type is poor except for adjusting to the local climate.
